# Accelerated telomere shortening independent of LRRK2 variants in Chinese patients with Parkinson's disease

**DOI:** 10.18632/aging.103878

**Published:** 2020-10-29

**Authors:** Yue Wu, Yuqing Pei, Zhuo Yang, Kexin Li, Xiaoying Lou, Wei Cui

**Affiliations:** 1 State Key Laboratory of Molecular Oncology, Department of Clinical Laboratory, National Cancer Center, National Clinical Research Center for Cancer, Cancer Hospital, Chinese Academy of Medical Sciences and Peking Union Medical College, Beijing 100021, China; 2 Department of Clinical Laboratory, Peking Union Medical College Hospital, Chinese Academy of Medical Sciences and Peking Union Medical College, Beijing 100730, China

**Keywords:** Parkinson’s disease, telomere length, aging, LRRK2 variants

## Abstract

Oxidative stress and inflammation play vital roles in Parkinson’s disease (PD) development. Thus, telomere length is expected to be shortened in this disease, but current data are inconclusive. We performed a case-control study of 261 patients with PD and 270 sex and age-matched healthy controls treated at the Peking Union Medical College Hospital. We found leucocyte telomere length (LTL) was significantly shortened in PD as compared with controls [1.02 (0.84-1.39) *vs*. 1.48 (1.08-1.94), P<0.001] and shorter LTL was associated with a dramatically increased risk of PD (lowest *vs*. highest quartile odds ratio (OR) =9.54, 95% CI: 5.33-17.06, P<0.001). We also investigated the roles of six LRRK2 variants in the susceptibility to PD. R1441C/G/H, G2019S, and I2020T variations were not detected in our study. No significant differences were found in the presence of variants R1398H (15.4% *vs*. 17.0%, P=0.619) and R1628P (2.3% *vs*. 0.7%, P=0.159) in PD and controls, while the G2385R variant was found to be a risk factor associated with increased PD susceptibility (OR=2.14, 95% CI: 1.12-4.10, P=0.021). No significant association was found between different LRRK2 variants and telomere length. These findings suggest that shorter LTL might be associated with PD in a manner independent of LRRK2 variants.

## INTRODUCTION

Parkinson’s disease (PD) is the second most common neurodegenerative disease affecting about 2% of the population aged over 60 years [1, 2]. A meta-analysis of available worldwide data showed a rising prevalence of PD with aging [3]. To face the social and economic burden along with the increasing number of PD patients, uncovering PD genetic biology and developing neuroprotective interventions are essential.

Telomeres are the repeated sequences that protect the ends of chromosomes and avoid cellular senescence and apoptosis induced by genomic instability [4]. The shortening of telomere length is regarded as an indicator of cellular aging, which is accelerated by oxidative stress and inflammation [5]. Mitochondrial dysfunction produces reactive oxygen species that can lead to oxidative damage, contributing to telomere shortening [6]. Many lines of evidence suggest that mitochondrial dysfunction plays a central role in the pathogenesis of PD [7]. In this pathological condition, telomere erosion may be accelerated. However, data on telomere shortening in Parkinson’s disease are inconsistent among various studies.

Leucine-rich repeat kinase 2 (LRRK2) is a multifunctional protein implicated in the regulation of various cellular functions [8]. Variants in LRRK2 have been identified as the most common candidate gene linked to both familial and sporadic Parkinson’s disease (PD) [9]. Several LRRK2 variants have been reported that affect the risk of PD, but data in Chinese patients are not always consistent. G2019S is the most common LRRK2 mutation with a high incidence in North African Arabic (39%) and Ashkenazi Jewish patients with PD (18.3%) [10, 11]. R1441C/G/H, three variations of the same codon, have been detected in several populations [12]. I2020T is identified in Japanese PD families, and is associated with increased kinase activity [13, 14]. G2385R and R1628P are two variants that are prevalent in Asian populations, also correlated with an increased risk of PD [15, 16]. However, the LRRK2 R1398H polymorphism is associated with a decreased risk of PD in a Han Chinese population [17]. Furthermore, LRRK2 variants are known to be associated with mitochondrial dysfunction. A single mutation in LRRK2 results in increased susceptibility to oxidative stressors, even though the mechanism behind it is not well understood [18].

We conducted this case-control study to investigate differences in the telomere length in a Chinese population. We also investigated the prevalence of three well-known pathogenic variants (R1441C/G/H, G2019S, I2020T) and three Asian-prevalent (R1398H, G2385R, R1628P) variants and assessed their roles in the susceptibility to PD. Furthermore, we analyzed the possible relationship between telomere length and LRRK2 variants.

## RESULTS

### General characteristics of subjects

**Figure 1:**
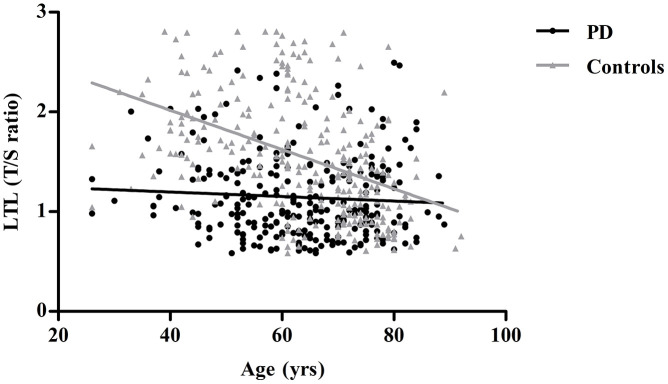
**Linear regression analysis of the association between leukocyte telomere length (LTL) and age in controls and PD patients.** Controls are shown as gray triangles (n=270) and PD patients as black dots (n=261).

Table 1 depicts the baseline characteristics of all participants. Significant shorter leukocyte telomere lengths (LTLs) were found in PD patients when compared with controls [1.02 (0.84-1.39) *vs*. 1.48 (1.08-1.94), P<0.001]. Moreover, the PD group had significantly lower levels of total protein (TP), albumin(Alb), total cholesterol (TC), triglycerides (TG), high-density lipoprotein cholesterol (HDL-C), and low-density lipoprotein cholesterol (LDL-C), higher level of homocysteine (Hcy). Telomere length was negatively correlated with age in controls, with a shorter LTL at higher age (r=-0.507, P<0.001), a relationship we did not find in the PD group (r=-0.073, P=0.239) (Figure 1). LTL did not differ significantly between males and females in any of the groups (data not shown).

**Table 1:** Clinical characteristics of patients with PD and control subjects.

**Clinical characteristics**	**Control subjects (n=270)**	**PD patients (n=261)**	**P value**
Age (years)	65 (55-74)	63 (54-74)	0.461
Male	148 (54.8%)	134 (51.3%)	0.423
LTL (T/S ratio)	1.48 (1.08-1.94)	1.02 (0.84-1.39)	**<0.001**
TP	72 (70-75)	71 (67-74)	**<0.001**
Alb	45 (44-46)	43 (41-46)	**<0.001**
TC (mmol/L)	4.78 ± 0.90	4.40 ± 1.02	**<0.001**
TG (mmol/L)	1.21 (0.87-1.56)	1.07 (0.75-1.45)	**0.02**
HDL-C (mmol/L)	1.35 (1.11-1.59)	1.17 (1.02-1.44)	**<0.001**
LDL-C (mmol/L)	3.13 ± 0.85	2.47 ± 0.79	**<0.001**
FBG (mmol/L)	5.3 (4.9-5.9)	5.4 (5.0-5.9)	0.182
hs-CRP (ng/mL)	0.67 (0.35-1.15)	0.79 (0.42-1.77)	0.277
Hcy (μmol/l)	13.5 (11.3-15.3)	15.3 (13.3-19.9)	**0.002**

Abbreviations: PD, Parkinson’s disease; LTL (T/S ratio), leukocyte telomere length (ratio of telomere repeat copy number (T) to single-copy gene copy number (S)); TP, Total protein; TC, Total cholesterol; TG, Triglyceride; HDL-C, High density lipoprotein cholesterol; LDL-C, Low density lipoprotein cholesterol; FBG, fasting blood glucose; hs-CRP, high-sensitivity C-reactive protein; Hcy, homocysteine.

Data are presented as n (%), mean ± standard deviation (SD), median (interquartile range). Significant P-values (P<0.05) are indicated in bold print.

### Short LTL increases the risk of PD

Using age and sex-adjusted logistic regression analyses, we investigated the relationship between PD and biochemical parameters which were significantly different between the groups, as shown in Table 1. Univariate logistic regression showed that higher TP, Alb, TC, HDL-C, and LDL-C were associated with a decreased risk of PD (Table 2). For LTL, we divided the patients into four groups based on the quartiles. A shorter LTL was associated with an increased risk of PD (lowest *vs*. highest quartile odds ratio (OR) =9.54, 95% CI: 5.33-17.06, P<0.001; P-value for the trend over quartiles: <0.001, Table 3). The results became more pronounced after multivariable-adjustment accounting for TP, Alb, TC, and HDL-C (lowest *vs*. highest quartile OR=75.23, 95% CI: 22.65-249.90, P<0.001; P-value for the trend over quartiles: <0.001, Table 3). Considering the high correlation between TC and LDL-C, LDL-C was not included in the multi-adjusted model.

**Table 2:** Logistic regression analyses of the association between clinical characteristics and PD adjusted for age and sex in all participants.

**Clinical characteristics**	**Age and sex adjusted**	
	**OR (95% CI)**	**P**
TG (mmol/L)	0.701 (0.484,1.016)	0.061
TP	0.924 (0.882,0.968)	**0.001**
Alb	0.842 (0.778,0.910)	**<0.001**
TC (mmol/L)	0.614 (0.480,0.787)	**<0.001**
HDL-C (mmol/L)	0.134 (0.062,0.293)	**<0.001**
LDL-C (mmol/L)	0.363 (0.268,0.492)	**<0.001**
Hcy (μmol/l)	1.021 (0.988,1.055)	0.21

Abbreviations: PD, Parkinson’s disease; TG, Triglyceride; TP, Total protein; Alb, Albumin; TC, Total cholesterol; HDL-C, High density lipoprotein cholesterol; LDL-C, Low density lipoprotein cholesterol; Hcy, homocysteine.

P-values and their ORs for the clinical parameters and PD were calculated by univariate logistic regression analysis. Significant P-values (P<0.05) are indicated in bold print.

**Table 3:** Logistic regression analyses of the association between LTL and PD in all participants.

**Clinical characteristics**	**Age and sex-adjusted model**			**Multivariable-adjusted model^a^**		
	**OR (95% CI)**	**P**	**P_trend_**	**OR (95% CI)**	**P**	**P_trend_**
LTL (T/S ratio)						
Q1	9.54 (5.33,17.06)	**<0.001**		75.23 (22.65,249.90)	**<0.001**	
Q2	5.53 (3.18,9.62)	**<0.001**		5.61 (2.44,12.91)	**<0.001**	
Q3	3.26 (1.89,5.64)	**<0.001**		3.40 (1.57,7.35)	**0.002**	
Q4	Reference	-	**<0.001**	Reference	-	**<0.001**

Abbreviations: PD, Parkinson’s disease; LTL (T/S ratio), ratio of telomere repeat copy number (T) to single-copy gene copy number (S).

^a^ Adjusted for age, sex, total protein, albumin, total cholesterol, high density lipoprotein cholesterol. Significant P-values (P<0.05) are indicated in bold print.

### Detecting LRRK2 variants in PD

Among six Asian-prevalent LRRK2 variants, the R1441C/G/H, G2019S, and I2020T variations were not detected in our study, indicating they may not be common pathogenic SNPs in the Chinese population. PD patients carried a higher frequency of variant G2385R than control subjects (11.2% *vs*. 5.5%; AA+AG *vs*. GG OR=2.14, 95% CI 1.12-4.10, P=0.021; A *vs*. G OR=2.05, 95% CI 1.11-3.82, P=0.023). However, no significant differences were found in the prevalence of variant R1398H (15.4% *vs*. 17.0%, P=0.619) and R1628P (2.3% *vs*. 0.7%, P=0.159) in patients with PD and healthy controls. The detailed information of this analysis is displayed in Table 4.

**Table 4:** Genotype and allele distribution of Asian-prevalent variants and the association with Parkinson’s disease.

**Genetic variants**	**Genotypes**	**Dominant model**				**Alleles**	**Allele model**			
		**Patients n=260**	**Controls n=271**	**OR (95% CI)**	**P value**		**Patients n=520**	**Controls n=542**	**OR (95% CI)**	**P value**
**R1398H**	AA	1	2	0.89 (0.56-1.41)	0.619	A	41	48	0.88 (0.57-1.36)	0.568
	AG	39	44			G	479	494		
	GG	220	225			-	-	-	-	-
**G2385R**	AA	1	1	2.14 (1.12-4.10)	**0.021**	A	30	16	2.05 (1.11-3.82)	**0.023**
	AG	28	14			G	490	526		
	GG	231	256			-	-	-	-	-
**R1628P**	CC	0	0	3.17 (0.64-15.89)	0.159	C	6	2	3.15 (0.63-15.69)	0.161
	CG	6	2			G	514	540		
	GG	254	269			-	-	-	-	-

### LRRK2 variants and telomere length

**Figure 2:**
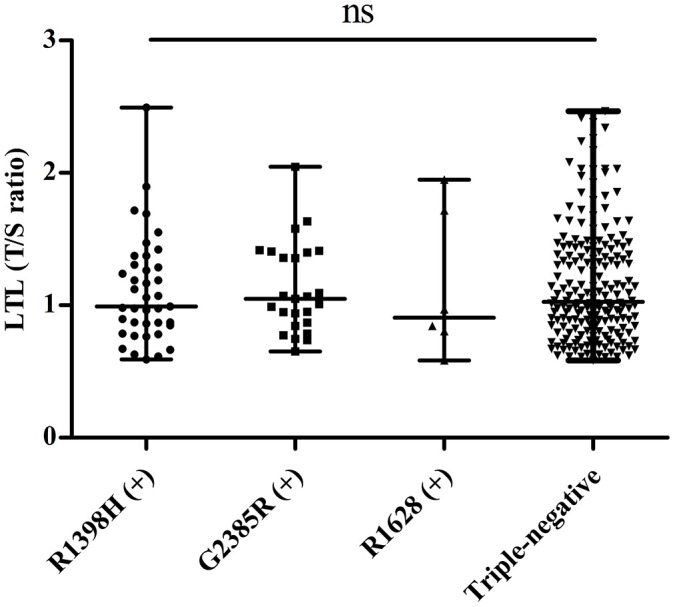
**Distribution of telomere length in PD patients with different LRRK2 variants.** “ns” means “not significant”.

To investigate whether LRRK2 variants affect telomere length, we divided PD patients into four groups: R1398H-positive, G2385R-positive, R1628P-positive, and triple-negative (indicating none of the three SNPs were positive). We excluded a patient carrying both R1398H and R1628P variants from further analysis. No significant differences were found among the four groups in age, sex, and LTL (Figure 2). The detailed information of this analysis is shown in Table 5.

**Table 5:** Clinical characteristics of PD patients with different LRRK2 variants.

**Clinical characteristics**	**G2385R- positive**	**R1398H- positive**	**R1628P- positive**	**Triple-negative**	**P value**
	**(n=25)**	**(n=39)**	**(n=6)**	**(n=190)**	
Age (years)	68 (63-77)	63 (52-74)	57 (46-69)	64 (54-73)	0.196
Male	18 (46.2%)	15 (60.0%)	4 (66.7%)	91 (47.9%)	0.574
LTL (T/S ratio)	1.05 (0.86-1.40)	0.99 (0.85-1.31)	0.91 (0.75-1.77)	1.03 (0.83-1.42)	0.943

Abbreviations: PD, Parkinson’s disease; LTL (T/S ratio), ratio of telomere repeat copy number (T) to single-copy gene copy number (S).

Data are presented as n (%), median (interquartile range).

## DISCUSSION

Our study found that patients with PD displayed shortened LTL, and shorter LTL increased the risk of PD dramatically. This was the first study investigating the relationship between telomere length and PD in Chinese patients. We also analyzed the six LRRK2 variants in PD. We demonstrated that G2385R is a risk factor associated with increased PD susceptibility in Chinese patients. However, we did not find any evidence that R1398H is a protective factor for PD, as other studies have reported. Furthermore, no significant relationship was found between LRRK2 variants and telomere length.

Both environmental factors and genetic predisposition affect LTL. The average telomere lengths of peripheral leukocytes are reported to become shorter with aging [19–21], while Hudson et al. [22] showed no correlation between telomere length and age in both PD cases and controls. In our study, a strong correlation was found between age and telomere shortening in all participants and controls, but not in the PD group. This may reflect other mechanisms beyond age that may be involved in telomere regulation, or some confounders such as mutation status, disease duration, and other biological markers in patients with PD may affect this relationship.

The shortening of telomeres is accelerated in various diseases characterized by oxidative stress and inflammation [23, 24]. Studies on telomere length in patients with PD were inconclusive. A meta-analysis, including eight primary studies, did not find consistent evidence of shorter telomeres comparing 959 patients and 1,284 controls. However, in our study, a significant shorter telomere length was found in patients with PD, and shorter LTL significantly increased the risk of PD. The discrepancy between different studies may be explained by variations in the study design, ethnicity, sample size, and age of the participants. Experimental data have shown that mice with short telomeres are characterized by a declined motor performance and an increased formation of α-synuclein aggregates that accelerate the progress of PD [25]. Additionally, CRISPR-Cas9-mediated telomere removal leads to mitochondrial dysfunction and PD-associated protein aggregation [26]. Thus, telomere shortening resulting from the inability to fully replicate the ends of linear chromosomes might also contribute to PD pathology. Another explanation is the decreased canonical Wnt signaling during PD development [27, 28]. Telomere protection is enhanced by the upregulation of Wnt/β-Catenin signaling [29, 30]. β-Catenin regulates Tert expression, thereby maintaining telomere length. Telomeres are also protected by the Wnt/β-catenin signaling pathway, which maintains TRF2 levels [29].

Multiple studies investigated the role of LRRK2 in the etiology of PD. LRRK2 has been implicated in mitochondrial dysfunction, Wnt signaling transduction, and protein translation control [31]. Over a hundred variants of the LRRK2 gene have been reported to date. Of these, G2019S, R1628P, G2385R, and R1398H have received much attention [32]. In our study, three pathogenic variants, R1441C/G/H, G2019S, and I2020T, which are common in western populations, were not identified. R1628P is a variant usually found in Asians, and although we detected it in some participants, no significant association was found with PD risk. A meta-analysis showed that east Asian individuals who harbored the R1398H variant had a 20% reduced risk of PD [33]. Our data found a similar but not significant trend, which may be due to the limited sample size. G2385R roughly doubled the risk of PD, which is consistent with other studies [33–35].

Considering the distinctive effects of these variants, we further compared the telomere length among the PD patients with different variants. Berwick et al. reported G2385R weakened Wnt signaling, while R1398H produced the opposite result [36]. This was also supported by a study by Jonathon et al. [37], who found that G2385R and R1398H play opposite roles regarding the effect of Wnt signaling. Thus, the idea that different variants may result in changes in telomere length is an attractive hypothesis. However, our data did not support this assumption. The limited sample size in our study may account for the negative result if the effect is weak. More extensive cohort studies and experimental data are needed to clarify the relationship between different LRRK2 variants and telomere length.

In conclusion, this is the first study investigating the relationship between telomere shortening and LRRK2 variants in patients with PD. Our findings indicate that a shorter LTL is associated with a dramatically increased risk of PD. G2385R is a risk factor associated with increased PD susceptibility in a Chinese population. No association was found between different LRRK2 variants and telomere length. These findings suggest that shorter LTL might be associated with PD in a manner independent of LRRK2 variants.

## MATERIALS AND METHODS

### Study design

We randomly recruited 261 PD patients (127 women, 134 men) from the Peking Union Medical College Hospital, China. All patients were diagnosed according to the UK PD Society Brain Bank criteria for clinical PD [38]. We recruited 270 sex and age-matched healthy controls (122 women, 148 men) who were visiting the hospital for a general health examination and did not have any neurological disorders. Participants with cancer, cardiovascular diseases, diabetes, hypertension, stroke, and current infections (defined as having a serum high-sensitivity C-reactive protein (hs-CRP) value of >10 mg/L) were excluded. All the participants were from the Chinese Han population. The study was approved by the Peking Union Medical College Hospital Ethics Committee and conformed to the Declaration of Helsinki principles. The requirement for written informed consent was waived by the institutional review board.

### Measurement of telomere length

Circulating leukocytes were collected, and DNA was extracted using the TIANamp Genomic DNA kit (Beijing). Telomere length was measured by a quantitative PCR method described by Cawthon et al. [39], which is based on the ratio of the telomere repeat copy number (T) to the single-copy gene copy number (S) expressed as the telomere length ratio (T/S ratio). The primers for telomere sequences used were: forward 5′-GGTTTTTGAGGGTGAGGG TGAGGGTGAGGGTGAGGGT-3′ and reverse 5′-TCCCGACTATCCCTATCCCTATCCCTATCCCTATCCCTA-3′. We used 36B4 as a single-copy reference gene, and the primers for that were: forward 5′-CAGCAAGT GGGAAGGTGTAATCC-3′ and reverse 5′-CCCATTCTATCATCAACGGGTACAA-3′. T/S ratios were expressed as LTL and were determined using the formula T/S = 2-^ΔCt^, where ΔCt = average Ct_Telomere_ - average Ct_36B4_. 293T cells were used for reference DNA samples, and we measured telomere length ratios by using a dilution series from 1.56 to 100.00 ng (2-fold dilution; 7 points) [40]. All samples were analyzed on the LightCycler 480 (Roche, Switzerland) and measured in triplicate.

### Clinical laboratory tests

Biochemical variables, including serum levels of TC, TG, HDL-C, and LDL-C were measured on a Beckman AU Series Automatic Biochemical Analyzer (Japan), using Sekisui Medical (Japan) reagents. Fasting blood glucose (FBG), high-sensitivity C-reactive protein (hs-CRP), TP, and Alb were measured with the same instrument, using Beckman AU reagents, and Hcy was examined using Beijing Leadman reagents.

### Genotyping

DNA samples were extracted from peripheral blood and amplified for sequence analysis. Three well-known pathogenic variants (R1441C/G/H, G2019S, I2020T) and three Asian-prevalent (R1398H, G2385R, R1628P) variants were genotyped to assess their roles in the susceptibility to PD. We applied sequence analysis to identify the six LRRK2 variants. The primers used for this method are shown in Supplementary Table 1.

### Statistics analysis

Continuous variables with a normal distribution were described as the mean ± standard deviation (SD) and analyzed by the unpaired t-test. In contrast, variables with a non-normal distribution are provided as the median (interquartile range) and were compared by the Mann-Whitney U or Kruskal Wallis Test. Sex was analyzed as percentages in the PD and control groups compared with the chi-square test. Linear regression was used to analyze the relationship between age and LTL. We used logistic regression to determine the risk of PD associated with each factor. The chi-square test was performed to compare the frequency distribution of genotypes and alleles, and the Hardy-Weinberg equilibrium was verified. Statistical significance was assumed at P < 0.05, and all analyses were conducted using SPSS 16.0 (SPSS Inc., Chicago, IL, USA).

## Supplementary Material

Supplementary Table 1

## References

[1] Tysnes OB, Storstein A. Epidemiology of Parkinson’s disease. J Neural Transm (Vienna). 2017; 124:901–05. 10.1007/s00702-017-1686-y 28150045

[2] Simon DK, Tanner CM, Brundin P. Parkinson disease epidemiology, pathology, genetics, and pathophysiology. Clin Geriatr Med. 2020; 36:1–12. 10.1016/j.cger.2019.08.002 31733690 PMC6905381

[3] Pringsheim T, Jette N, Frolkis A, Steeves TD. The prevalence of Parkinson’s disease: a systematic review and meta-analysis. Mov Disord. 2014; 29:1583–90. 10.1002/mds.25945 24976103

[4] Saretzki G. Telomeres, telomerase and ageing. Subcell Biochem. 2018; 90:221–308. 10.1007/978-981-13-2835-0_9 30779012

[5] Ahmed W, Lingner J. Impact of oxidative stress on telomere biology. Differentiation. 2018; 99:21–27. 10.1016/j.diff.2017.12.002 29274896

[6] Gonzales-Ebsen AC, Gregersen N, Olsen RK. Linking telomere loss and mitochondrial dysfunction in chronic disease. Front Biosci (Landmark Ed). 2017; 22:117–27. 10.2741/4475 27814605

[7] Winklhofer KF, Haass C. Mitochondrial dysfunction in Parkinson’s disease. Biochim Biophys Acta. 2010; 1802:29–44. 10.1016/j.bbadis.2009.08.013 19733240

[8] Alessi DR, Sammler E. LRRK2 kinase in Parkinson’s disease. Science. 2018; 360:36–37. 10.1126/science.aar5683 29622645

[9] Kluss JH, Mamais A, Cookson MR. LRRK2 links genetic and sporadic Parkinson’s disease. Biochem Soc Trans. 2019; 47:651–61. 10.1042/BST20180462 30837320 PMC6563926

[10] Lesage S, Dürr A, Tazir M, Lohmann E, Leutenegger AL, Janin S, Pollak P, Brice A, and French Parkinson’s Disease Genetics Study Group. LRRK2 G2019S as a cause of Parkinson’s disease in North African Arabs. N Engl J Med. 2006; 354:422–23. 10.1056/NEJMc055540 16436781

[11] Thaler A, Ash E, Gan-Or Z, Orr-Urtreger A, Giladi N. The LRRK2 G2019S mutation as the cause of Parkinson’s disease in Ashkenazi Jews. J Neural Transm (Vienna). 2009; 116:1473–82. 10.1007/s00702-009-0303-0 19756366

[12] Haugarvoll K, Rademakers R, Kachergus JM, Nuytemans K, Ross OA, Gibson JM, Tan EK, Gaig C, Tolosa E, Goldwurm S, Guidi M, Riboldazzi G, Brown L, et al. Lrrk2 R1441C Parkinsonism is clinically similar to sporadic Parkinson disease. Neurology. 2008; 70:1456–60. 10.1212/01.wnl.0000304044.22253.03 18337586 PMC3906630

[13] Funayama M, Hasegawa K, Ohta E, Kawashima N, Komiyama M, Kowa H, Tsuji S, Obata F. An LRRK2 mutation as a cause for the Parkinsonism in the original PARK8 family. Ann Neurol. 2005; 57:918–21. 10.1002/ana.20484 15880653

[14] Gloeckner CJ, Kinkl N, Schumacher A, Braun RJ, O’Neill E, Meitinger T, Kolch W, Prokisch H, Ueffing M. The Parkinson disease causing LRRK2 mutation I2020T is associated with increased kinase activity. Hum Mol Genet. 2006; 15:223–32. 10.1093/hmg/ddi439 16321986

[15] Tan EK, Tan LC, Lim HQ, Li R, Tang M, Yih Y, Pavanni R, Prakash KM, Fook-Chong S, Zhao Y. LRRK2 R1628P increases risk of Parkinson’s disease: replication evidence. Hum Genet. 2008; 124:287–88. 10.1007/s00439-008-0544-2 18781329

[16] Fu X, Zheng Y, Hong H, He Y, Zhou S, Guo C, Liu Y, Xian W, Zeng J, Li J, Liu Z, Chen L, Pei Z. LRRK2 G2385R and LRRK2 R1628P increase risk of Parkinson’s disease in a Han Chinese population from Southern mainland China. Parkinsonism Relat Disord. 2013; 19:397–98. 10.1016/j.parkreldis.2012.08.007 22981185

[17] Chen L, Zhang S, Liu Y, Hong H, Wang H, Zheng Y, Zhou H, Chen J, Xian W, He Y, Li J, Liu Z, Pei Z, Zeng J. LRRK2 R1398H polymorphism is associated with decreased risk of Parkinson’s disease in a Han Chinese population. Parkinsonism Relat Disord. 2011; 17:291–92. 10.1016/j.parkreldis.2010.11.012 21159540

[18] Puspita L, Chung SY, Shim JW. Oxidative stress and cellular pathologies in Parkinson’s disease. Mol Brain. 2017; 10:53. 10.1186/s13041-017-0340-9 29183391 PMC5706368

[19] Guan JZ, Maeda T, Sugano M, Oyama J, Higuchi Y, Suzuki T, Makino N. A percentage analysis of the telomere length in Parkinson’s disease patients. J Gerontol A Biol Sci Med Sci. 2008; 63:467–73. 10.1093/gerona/63.5.467 18511749

[20] Eerola J, Kananen L, Manninen K, Hellström O, Tienari PJ, Hovatta I. No evidence for shorter leukocyte telomere length in Parkinson’s disease patients. J Gerontol A Biol Sci Med Sci. 2010; 65:1181–84. 10.1093/gerona/glq125 20639300

[21] Maeda T, Guan JZ, Oyama J, Higuchi Y, Makino N. Age-related changes in subtelomeric methylation in the normal Japanese population. J Gerontol A Biol Sci Med Sci. 2009; 64:426–34. 10.1093/gerona/gln057 19223605 PMC2657167

[22] Hudson G, Faini D, Stutt A, Eccles M, Robinson L, Burn DJ, Chinnery PF. No evidence of substantia nigra telomere shortening in Parkinson’s disease. Neurobiol Aging. 2011; 32:2107.e3–5. 10.1016/j.neurobiolaging.2011.05.022 21794951 PMC4034165

[23] Valdes AM, Andrew T, Gardner JP, Kimura M, Oelsner E, Cherkas LF, Aviv A, Spector TD. Obesity, cigarette smoking, and telomere length in women. Lancet. 2005; 366:662–64. 10.1016/S0140-6736(05)66630-5 16112303

[24] Uziel O, Singer JA, Danicek V, Sahar G, Berkov E, Luchansky M, Fraser A, Ram R, Lahav M. Telomere dynamics in arteries and mononuclear cells of diabetic patients: effect of diabetes and of glycemic control. Exp Gerontol. 2007; 42:971–78. 10.1016/j.exger.2007.07.005 17709220

[25] Scheffold A, Holtman IR, Dieni S, Brouwer N, Katz SF, Jebaraj BM, Kahle PJ, Hengerer B, Lechel A, Stilgenbauer S, Boddeke EW, Eggen BJ, Rudolph KL, Biber K. Telomere shortening leads to an acceleration of synucleinopathy and impaired microglia response in a genetic mouse model. Acta Neuropathol Commun. 2016; 4:87. 10.1186/s40478-016-0364-x 27550225 PMC4994259

[26] Kim H, Ham S, Jo M, Lee GH, Lee YS, Shin JH, Lee Y. CRISPR-Cas9 mediated telomere removal leads to mitochondrial stress and protein aggregation. Int J Mol Sci. 2017; 18:2093. 10.3390/ijms18102093 28972555 PMC5666775

[27] Berwick DC, Harvey K. LRRK2 functions as a Wnt signaling scaffold, bridging cytosolic proteins and membrane-localized LRP6. Hum Mol Genet. 2012; 21:4966–79. 10.1093/hmg/dds342 22899650 PMC3709196

[28] Berwick DC, Harvey K. The regulation and deregulation of Wnt signaling by PARK genes in health and disease. J Mol Cell Biol. 2014; 6:3–12. 10.1093/jmcb/mjt037 24115276 PMC4344548

[29] Diala I, Wagner N, Magdinier F, Shkreli M, Sirakov M, Bauwens S, Schluth-Bolard C, Simonet T, Renault VM, Ye J, Djerbi A, Pineau P, Choi J, et al. Telomere protection and TRF2 expression are enhanced by the canonical Wnt signalling pathway. EMBO Rep. 2013; 14:356–63. 10.1038/embor.2013.16 23429341 PMC3615653

[30] Hoffmeyer K, Raggioli A, Rudloff S, Anton R, Hierholzer A, Del Valle I, Hein K, Vogt R, Kemler R. Wnt/β-catenin signaling regulates telomerase in stem cells and cancer cells. Science. 2012; 336:1549–54. 10.1126/science.1218370 22723415

[31] Verma M, Steer EK, Chu CT. ERKed by LRRK2: a cell biological perspective on hereditary and sporadic Parkinson’s disease. Biochim Biophys Acta. 2014; 1842:1273–81. 10.1016/j.bbadis.2013.11.005 24225420 PMC4016799

[32] Ross OA, Soto-Ortolaza AI, Heckman MG, Aasly JO, Abahuni N, Annesi G, Bacon JA, Bardien S, Bozi M, Brice A, Brighina L, Van Broeckhoven C, Carr J, et al, and Genetic Epidemiology Of Parkinson’s Disease (GEO-PD) Consortium. Association of LRRK2 exonic variants with susceptibility to Parkinson’s disease: a case-control study. Lancet Neurol. 2011; 10:898–908. 10.1016/S1474-4422(11)70175-2 21885347 PMC3208320

[33] Shu L, Zhang Y, Sun Q, Pan H, Tang B. A comprehensive analysis of population differences in LRRK2 variant distribution in Parkinson’s disease. Front Aging Neurosci. 2019; 11:13. 10.3389/fnagi.2019.00013 30760999 PMC6363667

[34] Xie CL, Pan JL, Wang WW, Zhang Y, Zhang SF, Gan J, Liu ZG. The association between the LRRK2 G2385R variant and the risk of Parkinson’s disease: a meta-analysis based on 23 case-control studies. Neurol Sci. 2014; 35:1495–504. 10.1007/s10072-014-1878-2 25027012

[35] Gopalai AA, Lim SY, Chua JY, Tey S, Lim TT, Mohamed Ibrahim N, Tan AH, Eow GB, Abdul Aziz Z, Puvanarajah SD, Viswanathan S, Looi I, Lim SK, et al. LRRK2 G2385R and R1628P mutations are associated with an increased risk of Parkinson’s disease in the Malaysian population. Biomed Res Int. 2014; 2014:867321. 10.1155/2014/867321 25243190 PMC4163406

[36] Berwick DC, Javaheri B, Wetzel A, Hopkinson M, Nixon-Abell J, Grannò S, Pitsillides AA, Harvey K. Pathogenic LRRK2 variants are gain-of-function mutations that enhance LRRK2-mediated repression of β-catenin signaling. Mol Neurodegener. 2017; 12:9. 10.1186/s13024-017-0153-4 28103901 PMC5248453

[37] Nixon-Abell J, Berwick DC, Grannó S, Spain VA, Blackstone C, Harvey K. Protective LRRK2 R1398H variant enhances GTPase and Wnt signaling activity. Front Mol Neurosci. 2016; 9:18. 10.3389/fnmol.2016.00018 27013965 PMC4781896

[38] Gibb WR, Lees AJ. The relevance of the lewy body to the pathogenesis of idiopathic Parkinson’s disease. J Neurol Neurosurg Psychiatry. 1988; 51:745–52. 10.1136/jnnp.51.6.745 2841426 PMC1033142

[39] Cawthon RM. Telomere measurement by quantitative PCR. Nucleic Acids Res. 2002; 30:e47. 10.1093/nar/30.10.e47 12000852 PMC115301

[40] Zhang DH, Wen XM, Zhang L, Cui W. DNA methylation of human telomerase reverse transcriptase associated with leukocyte telomere length shortening in hyperhomocysteinemia-type hypertension in humans and in a rat model. Circ J. 2014; 78:1915–23. 10.1253/circj.cj-14-0233 24882549

