# Supplementary material for: Accelerated telomere shortening independent of LRRK2 variants in Chinese patients with Parkinson's disease
Source: Aging (Albany NY). 2020 Oct 29;12(20):20483–92. doi: 10.18632/aging.103878 (PMC7655166; doi:10.18632/aging.103878)
Supplement: Supplementary Table 1 [file aging-12-103878-s001..pdf]

SUPPLEMENTARY TABLE

Supplementary Table 1. Primers for LRRK2 variants sequencing.

Names	Primer sequences 5'-3'
LRRK2-1628F	CTTCTAGGCCACATGGTTG
LRRK2-1628R	TCCTATTGGCAAAGCAATCT
LRRK2-2385F	AGCCCTGTTGTGGAAGTGT
LRRK2-2385R	AGAGGCAGAAAGGAAGAA
LRRK2-1398F	TAGGTACTTTGATCGGTTGCTGAC
LRRK2-1398R	GACTTCATTACTCGGAAAGTTTCCC
LRRK2-1441F	TCAACAGGAATGTGAGCAGG
LRRK2-1441R	CCCACAATTTTAAGTGAGTTGC
LRRK2-2019F	GATTTCCTGTGCATTTTCTGG
LRRK2-2019R	ACCTACCTGGTGTGCCCTCT
LRRK2-2020F	CAGATACCTCCACTCAGCC
LRRK2-2020R	TTTGACTCTTCTGAACTCACATC
